# Methylated DNA and high total DNA levels in the serum of patients with breast cancer following neoadjuvant chemotherapy are predictive of a poor prognosis

**DOI:** 10.3892/ol.2014.2068

**Published:** 2014-04-15

**Authors:** NORIKO FUJITA, NAOFUMI KAGARA, NORIAKI YAMAMOTO, KENZO SHIMAZU, ATSUSHI SHIMOMURA, MASAFUMI SHIMODA, NAOMI MARUYAMA, YASUTO NAOI, KOJI MORIMOTO, NAOFUMI ODA, SEUNG JIN KIM, SHINZABURO NOGUCHI

**Affiliations:** 1 Department of Breast and Endocrine Surgery, Osaka University Graduate School of Medicine, Suita-shi, Osaka 565-0871, Japan; 2 Central Research Laboratories, Sysmex Corporation, Kobe 651-2271, Japan

**Keywords:** breast cancer, neoadjuvant chemotherapy, methylation, DNA, serum, prognosis

## Abstract

In a previous study, we established a one-step methylation-specific polymerase chain reaction (OS-MSP) assay for the detection of methylated DNA (met-DNA) and total DNA levels in serum. For the present study, this OS-MSP assay was used for patients with breast cancer treated with neoadjuvant chemotherapy (NAC) in order to investigate the prognostic significance of met-DNA and total DNA levels. Following treatment with NAC and prior to surgery, serum samples obtained from 120 patients with stage II/III breast cancer were subjected to the OS-MSP assay for analysis of the glutathione S-transferase pi 1, Ras association (RalGDS/AF-6) domain family member 1 and retinoic acid receptor β2 genes. The detection of methylation in a minimum of one of these genes indicated a positive outcome of the assay. The total DNA content of the serum was also determined. Of the 120 stage II/III patients, seven (6%) were positive for met-DNA in serum and showed a significantly worse overall survival (OS) time compared with patients negative for met-DNA (n=113) (5-year OS, 43 vs. 85%; P=0.002). The patients with high total DNA levels in serum (n=40) also showed a significantly worse OS compared with those with low total DNA levels (n=80) (65 vs. 91%; P<0.001). The presence of met-DNA and high total DNA levels in the serum were found to be significant prognostic factors that are independent of a pathological complete response by multivariate analysis. Following NAC, met-DNA and high total DNA levels in the serum detected with the OS-MSP assay constitute novel prognostic factors for patients with breast cancer; this may be clinically useful for the prognosis prediction for patients who do not achieve a pathological complete response following NAC.

## Introduction

In recent years, growing numbers of patients with breast cancer have been treated with neoadjuvant chemotherapy (NAC) to shrink the tumor size for an improved chance of breast conservation. In fact, it has been reported by specific studies that more patients treated with NAC undergo breast-conserving surgery than those not treated (1,2). Another advantage of NAC is that the patient prognosis can be estimated by means of a pathological evaluation of the surgical specimens, i.e., if a pathological complete response (pCR) is achieved, the patient prognosis can be expected to be excellent, while the prognosis of patients who do not achieve a pCR is reportedly worse, with a 5-year relapse-free survival rate of 50–70% (3,4). Since prognostic evaluation is extremely important in deciding whether or not further adjuvant therapy should be used, more effective prognostic factors are required for those patients who cannot achieve a pCR.

It has been hypothesized and is currently accepted that the detection of tumor-specific DNA methylation in serum is useful for prognosis prediction and for monitoring responses to systemic therapy in patients with breast cancer (5–13). Certain studies have assessed the prognostic value of the presence of methylated DNA (met-DNA) in the serum of patients with breast cancer (5–9). It has also been reported that breast cancer patients with met-DNA in the serum detected by one-step methylation-specific polymerase chain reaction (PCR) show a poorer prognosis compared with those without it (10,11). However, other studies have reported that met-DNA in the serum of patients with breast cancer treated with adjuvant therapy correlates with the pathological response (12,13). A study by Sharma *et al* (12) detected methylation of the glutathione S-transferase pi 1 (*GSTP1*) and breast cancer 1, early onset (*BRCA1*) genes in serum more frequently in non-responders to NAC than in responders, while Avraham *et al* (13) reported that none of the responders to NAC showed methylated Ras association (RalGDS/AF-6) domain family member 1 (*RASSF1A*) in the serum. However, no studies have been published on the correlation between met-DNA in serum and the prognosis for patients with breast cancer treated with NAC. Therefore, the present study investigated whether the presence of met-DNA in the serum, as detected by one-step methylation-specific PCR (OS-MSP), may be associated with a poor prognosis for patients with breast cancer treated with NAC. In addition, the prognostic significance of high total DNA levels in the serum was also investigated.

## Materials and methods

### Patients

Patients with invasive breast cancer (n=120) who underwent breast conserving surgery or mastectomy following NAC at the Osaka University Hospital (Suita-shi, Osaka, Japan) between March 2000 and May 2007 were retrospectively included in the present study. Informed consent was obtained from each patient. A total of 44 patients had been treated with paclitaxel (80 mg/m^2^) weekly for 12 cycles followed by 5-fluorouracil (500 mg/m^2^), epirubicin (75 mg/m^2^) and cyclophosphamide (500 mg/m^2^) every three weeks for 4 cycles (P-FEC). Another 37 patients had been treated with docetaxel (75 mg/m^2^) every three weeks for 4 cycles, while 29 had been treated with cyclophosphamide (600 mg/m^2^) and epirubicin (60 mg/m^2^) every three weeks for 4 cycles, followed by docetaxel (60 mg/m^2^) every three weeks for 4 cycles. Finally, 10 patients had been treated with other types of chemotherapy consisting of docetaxel (60 mg/m^2^) or cyclophosphamide (600 mg/m^2^) and epirubicin (60 mg/m^2^) every three weeks or paclitaxel (80 mg/m^2^) weekly.

Serum samples were obtained following NAC and prior to surgery, and stored at −80°C until use. The median follow-up period was 73 months (range, 3–134 months) and the median age of the patients at the time of surgery was 51 years (range, 26–75 years). The clinicopathological characteristics of the patients are summarized in Table I. Adjuvant hormonal therapy was administered to 79 patients: Tamoxifen for 27, goserelin plus tamoxifen for 13 and anastrozole for 39 patients, all essentially in accordance with the St. Gallen recommendations (14–16). Subsequent to the surgery, the patients were followed up every 3 months for 1–2 years, every 6 months for 3–5 years and once every year thereafter. This study was approved by the Ethics Committee of Osaka University Graudate School of Medicine (Suita, Osaka, Japan).

### OS-MSP assay for GSTP1, RASSF1A and retinoic acid receptor β2 (RARβ2) promoter hypermethylation in serum

The OS-MSP assay was conducted as previously described (10). In brief, a total of 1,000 μl of serum from each patient was solubilized by incubation with lysis buffer (5 M guanidine-HCl and 0.65 mg/ml proteinase K) at 50°C for 1 h and then incubated with a 5 M bisulfite solution at 80°C for 40 min. Bisulfite-modified DNA was purified with a DNA purification kit (Qiagen, Valencia, CA, USA) and eluted in 50 μl dH_2_O. Modification by bisulfite was completed by treatment with 0.3 M NaOH for 5 min at room temperature, subsequent to which bisulfite-modified DNA was extracted by gel filtration (GE Healthcare, Princeton, NJ, USA) for use as the PCR template. Promoter hypermethylation of *GSTP1, RASSF1A* and *RARβ*2 was then evaluated by OS-MSP. The primer and probe sequences were as follows: *GSTP1* forward primer, 5′-CGTCGTGATTTAGTATTGGGGC-3′ and reverse primer, 5′-CTAATAACGAAAACTACGACGACGAAA-3′; *GSTP1* probe, 5′-FAM-ATAAGGTTCGGAGGTCGCGAGGTTTTC GT-DDQ1-3′; *RASSF1A* forward primer, 5′-ATAGTTTTTGTA TTTAGGTTTTTATTGCGC-3′ and reverse primer, 5′-GCT AACAAACGCGAACCG-3′; *RASSF1A* probe, 5′-FAM-TTG AAGTCGGGGTTCGTTTTGTGGTTTCGT-DDQ1-3′; *RARβ2* forward primer, 5′-GAATATCGTTTTTTAAGTTAAGTC GTC-3′ and reverse primer, 5′-GAAACGCTACTCCTAACT CACG-3′; and *RARβ2* probe, 5′-FAM-AGGCGTAAAGGG AGAGAAGTTGGTGTTTA-DDQ1-3′. For the PCR amplifications, the Light Cycler 480 Real-Time PCR System (Roche Applied Science, Madison, WI, USA) was used under the following conditions: 1 cycle at 95°C for 10 min, followed by 50 cycles of 95°C for 30 sec, 60°C for 30 sec and 72°C for 30 sec. Every sample was analyzed in a single assay for each gene. Finally, the PCR products were also analyzed by means of 3% agarose gel electrophoresis, staining with ethidium bromide and visualization under UV illumination.

### Assay for total DNA levels in serum

Total DNA levels were quantified as previously described (10). In brief, in order to quantify total DNA levels in serum following bisulfite treatment, a genomic locus lacking a cytosine base was selected, since such a locus is not affected by bisulfite treatment. This locus was amplified by PCR using the primers, probe and standard oligoDNA. The primer and probe sequences were as follows: Forward primer, 5′-AGGGAGTAGAGAAAAAGT AGGAAGATGAGT-3′ and reverse primer, 5′-TCCAACATC ACATCCAATCCA-3′; probe, 5′-FAM-AGGGTGATAATG AGTGTGTTGGGAAATAGA-DDQ1-3′; and standard oligo DNA, 5′-AGGGAGTAGAGAAAAAGTAGGAAGATGAGT CCAGGGTGATAATGAGTGTGTTGGGAAATAGACCTG GATTGGATGTGATGTTGGA-3′. The PCR conditions were as aforementioned. The patients were divided into three tertiles (low, middle and high) according to the level of total DNA in serum. Those in the low and middle tertiles were then combined and treated as the low total DNA group and those in the high tertile were treated as the high total DNA group.

### DNA extraction from tumor tissues

DNA was also extracted as previously described (17) from breast tumor tissues obtained prior to (core-needle biopsy specimens) or following (surgical specimens) NAC from the patients who showed positive results for the OS-MSP assay in at least one of the three genes. The specimens were then examined as to whether the corresponding genes were methylated in the tumor tissues. For this examination, 1 μg of DNA was subjected to sodium bisulfite treatment using the EpiTect Bisulfite kit (48; Qiagen) according to the manufacturer’s instructions, and analyzed by the OS-MSP assay for promoter hypermethylation of *GSTP1, RASSF1A* and *RARβ2,* as previously described (10).

### Histological grade and estrogen receptor (ER), progesterone receptor (PR) and human epidermal growth factor receptor 2 (HER2) expression

Histological grade was determined using the Scarff-Bloom-Richardson grading system (18). ER and PR were classified as positive when ≥10% of the tumor cells showed immunohistochemically-positive staining (ER, clone 6F11; and PR, clone 16; Ventana Japan K.K., Yokohama and SRL, Inc., Tokyo, Japan, respectively). HER-2 expression was determined immunohistochemically using anti-human c-erbB-2 polyclonal antibody (Nichirei Biosciences, Inc., Tokyo, Japan) or by means of fluorescence *in situ* hybridization (FISH) using PathVysion Her2 DNA Probe kits (SRL, Inc.). When a tumor showed +3 immunostaining (positive tumor cells >30%) or a FISH ratio (HER2 gene signals to chromosome 17 centromere signals) of ≥2.0, it was considered HER2-positive.

### Evaluation of the response to chemotherapy

The pathological response to chemotherapy was evaluated by examining the surgical specimens obtained during the surgery. The specimens were cut at 5-mm intervals for the preparation of hematoxylin and eosin sections. A complete loss of invasive tumor cells in the primary tumor site without any lymph node metastasis was classified as a pCR.

### Statistical analysis

Associations of met-DNA or total DNA levels in serum with various clinicopathological parameters were assessed by means of Pearson’s χ^2^ test. Disease-free survival (DFS) was calculated as the time from surgery until the date of any recurrence of breast cancer (local or distant) or mortality from any cause. Overall survival (OS) was calculated as the time from surgery to the date of mortality from any cause. DFS and OS were assessed with the Kaplan-Meier method and log-rank tests, while univariate and multivariate analyses (Cox regression models) were used to evaluate the prognostic significance of various parameters. Multivariate analyses included the parameters P<0.1 or known prognostic factor (pCR). All the statistical analyses were two-sided and P<0.05 was considered to indicate a statistically significant difference. SPSS software (SPSS, Inc., Chicago, IL, USA) was used for all statistical analyses.

## Results

### Associations between clinicopathological parameters and met-DNA or total DNA levels in serum

Promoter hypermethylation of *GSTP1, RASSF1A* and *RARβ2* in serum was detected with the OS-MSP assay in 4, 1 and 2% of the 120 patients, respectively. When promoter hypermethylation was found in at least one of these three genes, the result of the OS-MSP assay for met-DNA in serum was considered to be positive for the subsequent analysis. A total of seven patients (6%) were found to be positive for met-DNA in serum. No significant association was observed between met-DNA in serum and any clinicopathological characteristics (Table I). The promoter methylation status of the genes methylated in the serum of the seven patients who were positive for met-DNA was also examined in the tumor specimens obtained from the same patients. The same genes that were methylated in the serum were also found to be methylated in the tumor tissue of each of the seven patients.

The median total DNA level in the serum was 1.9 ng/ml (range, 0–63.3 ng/ml). The patients were divided into tertiles (high, middle and low) according to the level of total DNA in the serum. The middle and low tertiles were combined and treated as the low total DNA group (n=80), and the high tertile was treated as the high total DNA group (n=40). The cut-off value for the high and low total DNA groups was 3.3 ng/ml. Correlations between the total DNA levels in the serum and the clinicopathological characteristics are shown in Table I. The patients in the high total DNA group were significantly more likely to have tumors of high histological grade (52 vs. 29%, P=0.031) and to be positive for met-DNA in the serum (71 vs. 31%, P=0.028). By contrast, pCR was not significantly associated with either met-DNA or total DNA levels in the serum.

### Association of prognosis with met-DNA or total DNA levels in serum

**Figure 1:**
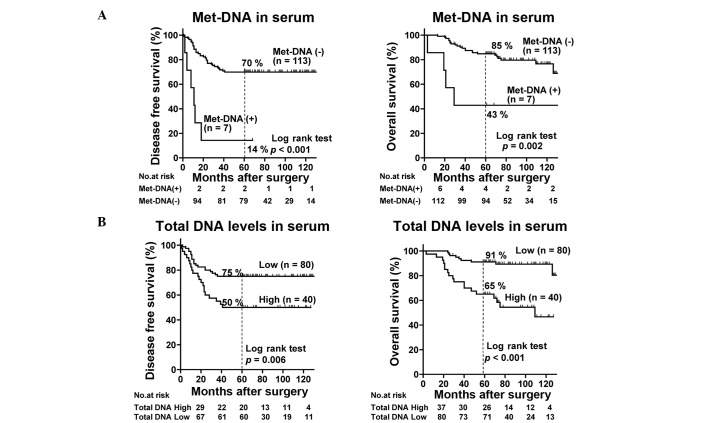
Prognosis of NAC-treated breast cancer patients in association with (A) met-DNA or (B) total DNA levels in the serum. Disease-free survival (DFS) and overall survival (OS) rates were evaluated in all the NAC-treated patients in association with the presence of met-DNA and total DNA levels in the serum. NAC, neoadjuvant chemotherapy; Met-DNA (−), patients negative for methylated DNA in the serum; Met-DNA (+), patients positive for methylated DNA in the serum; high, patients in the high tertile of total DNA; low, combination of patients in the low and middle tertiles of total DNA.

The seven patients with serum positive for met-DNA exhibited significantly worse DFS and OS rates compared with those negative for met-DNA (n=113) (P<0.001 and P=0.002, respectively; Fig. 1A), while the patients with high total DNA levels in the serum (n=40) also showed significantly worse DFS and OS rates compared with those with low levels of total DNA (n=80) (P=0.006 and P<0.001, respectively; Fig. 1B). Subsequently, univariate and multivariate analyses were conducted to determine whether met-DNA and total DNA levels in the serum are independent prognostic factors for the other parameters (Tables II and III). The multivariate analysis showed that met-DNA in the serum was significantly associated with DFS (P=0.003) and OS (P=0.009), as was total DNA levels in the serum (P=0.045 and P=0.001, respectively).

**Figure 2:**
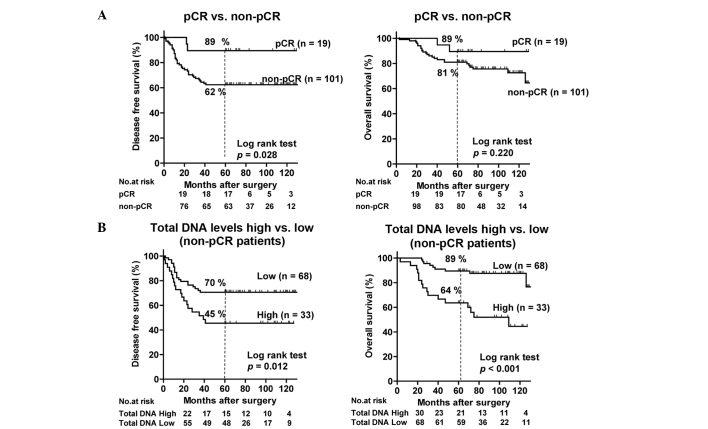
Prognosis of NAC-treated breast cancer patients in association with the pathological response. Disease-free survival (DFS) and overall survival (OS) rates were assessed for (A) NAC-treated patients (n=120) according to pathological response and (B) NAC-treated patients (n=101) without pCR according to total DNA levels in the serum. NAC, neoadjuvant chemotherapy; pCR, pathological complete response; high, patients in the high tertile of total DNA in the serum; low, combination of patients in the low and middle tertiles of total DNA in the serum.

The patients who achieved pCR (n=19) showed significantly improved DFS rates compared with those who did not (n=101) (P=0.028; Fig. 2A), and the prognostic significance for the latter group of total DNA levels in the serum was evaluated. The patients with high total DNA levels in the serum (n=33) again showed significantly worse DFS and OS rates compared with those with low total DNA levels (n=68) (P=0.012 and P<0.001, respectively; Fig. 2B).

## Discussion

The present study investigated whether detection of met-DNA and high total DNA levels in the serum by means of the OS-MSP assay could serve as novel prognostic factors for patients with breast cancer treated with NAC. By using multivariate analysis it was shown that met-DNA and total DNA levels in the serum following NAC are independent prognostic factors for DFS and OS, independent of pCR, which is a well-established prognostic factor for NAC-treated patients. The results indicate that met-DNA and total DNA levels in the serum may be clinically useful prognostic factors.

Met-DNA is believed to represent circulating tumor genomes, but not necessarily total DNA levels, as it can not only originate from tumor cells, but also from inflammatory cells, endothelial cells and fibroblasts in tumor tissues (19–21). Although the reason why total DNA levels in the serum is associated with prognosis remains unclear, it is possible that high total DNA levels in the serum may reflect specific tumor biology that is associated with tumor metastasis and tumor-induced inflammation. It is also possible that circulating tumor cells (22,23) and micrometastatic deposits of distant organs, including the bone marrow and liver, may contribute to total DNA levels (19,24).

The OS-MSP assay showed that the serum was positive for met-DNA in 6% of patients in the present study. This positivity appears to be slightly lower compared with our previous study, in which it was found that met-DNA was positively detected in 10% of stage I and II patients without NAC treatment (11). These results indicate that positivity of met-DNA in serum decreases following NAC. In fact, studies by Sharma *et al* (12) and Avraham *et al* (13) reported a significant decline in met-DNA levels following NAC. However, in the present study, the serum samples were not obtained prior to NAC and thus the change in the positivity of met-DNA prior to and following NAC could not be investigated.

While a significant correlation between met-DNA and pCR was not detected, none of the patients that were positive for met-DNA achieved pCR, which is consistent with the findings of other studies (12,13). Sharma *et al* (12) reported that all non-responders were positive for met-DNA (*GSTP1* and *BRCA1*) following NAC, and Avraham *et al* (13) reported that all patients that were positive for met-DNA (*RASSF1A*) following NAC could not achieve pCR. These results appear to indicate that positivity for met-DNA following NAC is associated with non-pCR.

A limitation of the present study is that the serum samples could be obtained following, but not prior to, NAC, so that the prognostic significance of met-DNA and total DNA levels in serum prior to NAC could not be addressed. However, the available data appears to indicate that met-DNA following NAC is a highly significant prognostic indicator for poor prognosis, as none of the patients that were positive for met-DNA attained pCR, and as many as 86% developed recurrence. High total DNA levels in the serum following NAC were also significantly associated with a poor prognosis. It has been reported that the quantity of disseminated or circulating tumor cells detected following, but not prior to, chemotherapy can predict prognosis, as the numbers detected following chemotherapy may reflect the residual presence of micro-metastatic tumors (25,26). In analogy to this finding, the presence of met-DNA and high total DNA levels may represent the residual presence of micro-metastatic tumors. Patients with met-DNA and/or high total DNA levels in the serum can thus be considered to be essentially resistant to NAC and to have a poor prognosis, necessitating the use of non-cross resistant, post-operative adjuvant chemotherapy.

In conclusion, the present study was able to demonstrate that the presence of met-DNA and high levels of total DNA detected with the OS-MSP assay is a significant and independent prognostic factor for patients with breast cancer treated with NAC. The OS-MSP assay may thus be clinically useful for the selection of NAC-treated patients who are at a high risk of relapse and thus require treatment with additional post-operative adjuvant chemotherapy. To the best of our knowledge, this is the first study to assess the association between met-DNA or total DNA levels in the serum and prognosis following NAC. However, future studies including larger numbers of patients with sufficient follow-up times are required to further validate the clinical utility of the OS-MSP assay for patients treated with NAC.

## Figures and Tables

**Table I:** Correlations between the presence of methylated DNA or levels of total DNA in serum and the clinicopathological parameters prior to NAC^a^.

		Met-DNA^b^ in serum			Total DNA levels in serum		
			
	n	Met (−)^c^, n (%)	Met (+)^d^, n (%)	P-value^e^	Low, n (%)	High, n (%)	P-value^f^
Total patients	120	113 (94)	7 (6)	80 (67)	40 (33)		
Menopausal status							
Premenopausal	62	60 (97)	2 (3)	0.261	44 (71)	18 (29)	0.301
Postmenopausal	58	53 (91)	5 (9)		36 (62)	22 (38)	
Tumor size							
T1	3	3 (100)	0 (0)	0.424^g^	2 (67)	1 (33)	0.763^g^
T2	71	68 (96)	3 (4)		47 (66)	24 (34)	
T3	22	21 (95)	1 (5)		15 (68)	7 (32)	
T4	23	20 (87)	3 (13)		16 (70)	7 (30)	
Unknown	1	1 (100)	0 (0)		0 (0)	1 (100)	
Lymph node metastasis							
N0	35	35 (100)	0 (0)	0.105	24 (69)	11 (31)	0.776
N1	85	78 (92)	7 (8)		56 (66)	29 (34)	
Histological grade							
G1	26	26 (100)	0 (0)	0.116^h^	20 (77)	6 (23)	0.031^h^
G2	64	61 (95)	3 (5)		44 (69)	20 (31)	
G3	25	22 (88)	3 (12)		12 (48)	13 (52)	
Unknown	5	4 (80)	1 (20)		4 (80)	1 (20)	
Histological type							
Invasive ductal carcinoma	111	106 (95)	5 (5)	0.125	73 (66)	38 (34)	0.811^e^
Invasive lobular carcinoma	8	6 (75)	2 (25)		6 (75)	2 (25)	
Others	1	1 (100)	0 (0)		1 (100)	0 (0)	
ER							
Negative	53	48 (91)	5 (9)	0.239	35 (66)	18 (34)	0.897
Positive	67	65 (97)	2 (3)		45 (67)	22 (33)	
PR							
Negative	72	66 (92)	6 (8)	0.240	46 (64)	26 (36)	0.429
Positive	48	47 (98)	1 (2)		34 (71)	14 (29)	
HER2							
Negative	88	83 (94)	5 (6)	1.000	61 (69)	27 (31)	0.398
Positive	28	27 (96)	1 (4)		17 (61)	11 (39)	
Unknown	4	3 (75)	1 (25)		2 (50)	2 (50)	
CEA							
Negative	103	97 (94)	6 (6)	1.000	70 (68)	33 (32)	0.263^d^
Positive	15	14 (93)	1 (7)		8 (53)	7 (47)	
Unknown	2	2 (100)	0 (0)		2 (100)	0 (0)	
CA15-3							
Negative	107	101 (94)	6 (6)	0.505	71 (66)	36 (34)	1.000^d^
Positive	11	10 (91)	1 (9)		7 (64)	4 (36)	
Unknown	2	2 (100)	0 (0)		2 (100)	0 (0)	
Pathological response							
Non-pCR	101	94 (93)	7 (7)	0.595	68 (67)	33 (33)	0.724
pCR	19	19 (100)	0 (0)		12 (63)	7 (37)	

a Neoadjuvant chemotherapy;

b methylated DNA (met-DNA);

c negative for met-DNA in serum;

d positive for met-DNA in serum;

e Fisher’s exact test;

f χ^2^ test;

g T1 + T2 vs. T3 + T4;

h G1 + G2 vs. G3.

NAC, neoadjuvant chemotherapy; ER, estrogen receptor; PR, progesterone receptor; HER2, human epidermal growth factor receptor 2; CEA, carcinoembryonic antigen; CA, cancer antigen; pCR, pathological complete response.

**Table II:** Univariate and multivariate analysis of various prognostic factors for DFS of 120 patients.

	Univariate analysis			Multivariate analysis		
		
Parameters	HR	95% CI	P-value	HR	95% CI	P-value
Met-DNA in serum (positive vs. negative)	6.650	2.74–16.17	<0.001	4.226	1.63–10.95	0.003
Total DNA levels in serum (high vs. low)	2.336	1.26–4.34	0.007	1.926	1.02–3.654	0.045
Tumor size (3,4 vs. 1,2) prior to NAC	1.422	0.76–2.68	0.276			
Histological grade (3 vs. 1,2) prior to NAC	1.560	0.76–3.21	0.227			
LN (positive vs. negative) prior to NAC	2.856	1.20–6.81	0.018	2.864	1.17–7.01	0.021
CEA (positive vs. negative) prior to NAC	2.050	0.94–4.45	0.070	2.074	0.94–4.59	0.072
CEA (positive vs. negative) following NAC	1.815	0.56–5.89	0.321			
CA15-3 (positive vs. negative) prior to NAC	1.099	0.39–3.09	0.858			
CA15-3 (positive vs. negative) following NAC	0.433	0.06–3.15	0.408			
NAC (P-FEC vs. others)	0.528	0.26–1.08	0.080	0.589	0.28–1.24	0.163
Pathological response (pCR vs. non-pCR)	0.234	0.06–0.97	0.045	0.260	0.06–1.12	0.071

DFS, disease-free survival; HR, hazard ratio; CI, confidence interval; Met-DNA, methylated DNA; NAC, neoadjuvant chemotherapy; LN, lymph node metastasis; CEA, carcinoembryonic antigen; CA, cancer antigen; P-FEC, paclitaxel 80 mg/m^2^ for 12 cycles followed by 5-fluorouracil (500 mg/m^2^), epirubicin (75 mg/m^2^) and cyclophosphamide (500 mg/m^2^) every three weeks for 4 cycles; pCR, pathological complete response.

**Table III:** Univariate and multivariate analysis of various prognostic factors for OS of 120 patients.

	Univariate analysis			Multivariate analysis		
		
Parameters	HR	95% CI	P-value	HR	95% CI	P-value
Met-DNA in serum (positive vs. negative)	4.834	1.65–14.15	0.004	4.805	1.49–15.52	0.009
Total DNA levels in serum (high vs. low)	5.119	2.28–11.49	<0.001	4.112	1.77–9.57	0.001
Tumor size (3,4 vs. 1,2)	1.366	0.63–2.97	0.430			
Histological grade (3 vs. 1,2) prior to NAC	2.530	1.16–5.53	0.020	2.302	1.02–5.21	0.046
LN (positive vs. negative) prior to NAC	2.179	0.82–5.77	0.117			
CEA (positive vs. negative) prior to NAC	2.298	0.93–5.70	0.073	3.497	1.34–9.11	0.010
CEA (positive vs. negative) following NAC	0.643	0.09–4.75	0.666			
CA15-3 (positive vs. negative) prior to NAC	2.087	0.72–6.04	0.175			
CA15-3 (positive vs. negative) following NAC	0.046	0.00–65.53	0.405			
NAC (P-FEC vs. others)	0.744	0.31–1.80	0.510			
Pathological response (pCR vs. non-pCR)	0.418	0.10–1.77	0.235	0.258	0.06–1.18	0.081

OS, overall survival; HR, hazard ratio; CI, confidence interval; Met-DNA, methylated DNA; NAC, neoadjuvant chemotherapy; LN, lymph node metastasis; CEA, carcinoembryonic antigen; CA, cancer antigen; P-FEC, paclitaxel 80 mg/m^2^ for 12 cycles followed by 5-fluorouracil (500 mg/m^2^), epirubicin (75 mg/m^2^) and cyclophosphamide (500 mg/m^2^) every three weeks for 4 cycles; pCR, pathological complete response.
